# Supplementary material for: Triptonide is a reversible non-hormonal male contraceptive agent in mice and non-human primates
Source: Nat Commun. 2021 Feb 23;12:1253. doi: 10.1038/s41467-021-21517-5 (PMC7902613; doi:10.1038/s41467-021-21517-5)
Supplement: Supplementary file 1 — Supplementary Information [file 41467_2021_21517_MOESM1_ESM.pdf]

Supplementary Information

Triptonide Is A Reversible, Non-Hormonal Male Contraceptive Agent in Male Mice and Non-Human Primates

Zongliang Chang[1,*], Weibing Qin[2,3,*], Huili Zheng[1,*], Kathleen Schegg[1,*], Lu Han[2,3], Xiaohua Liu[2,3], Yue Wang[1], Zhuqing Wang[1], Hayden McSwiggin[1], Hongying Peng[1], Shuiqiao Yuan[1], Jiabao Wu[2,3], Yongxia Wang[2,3], Shenghui Zhu[2,3], Yanjia Jiang[2,3], Hua Nie[2,3], Yuan Tang[2,3], Yu Zhou[2,3], Michael J. M. Hitchcock[4], Yunge Tang[2,3] and Wei Yan[1,5,6]

[1]Department of Physiology and Cell Biology, University of Nevada, Reno School of Medicine, Reno, NV 89557, USA. [2]NHC Key Laboratory of Male Reproduction and Genetics, Guangzhou 510600, P. R. China. [3]Family Planning Research Institute of Guangdong Province, Guangzhou 510600, P. R. China. [4]Department of Microbiology and Immunology, University of Nevada, Reno School of Medicine, Reno, NV 89557, USA. [5]The Lundquist Institute for Biomedical Innovation at Harbor-UCLA Medical Center, Torrance, CA 90502, USA. [6]Department of Medicine, David Geffen School of Medicine at UCLA, Los Angeles, CA 90095, USA.

*These authors contributed equally to this work (ZC, WQ, HZ and KS)

The supplementary information contains 16 figures (Figs.1-16) and 4 tables (Tables 1-4).

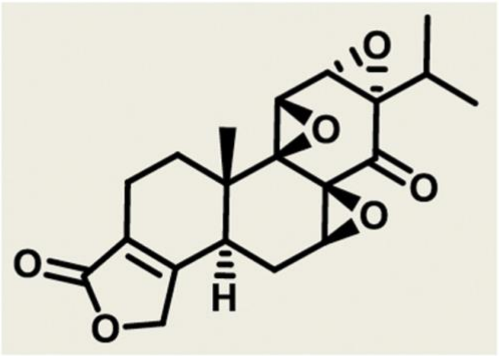

($C_{20}H_{22}O_6$, M.W.=358)

Supplementary Fig. 1 Structure and molecular weight of triptonide, a natural compound purified from the herb *Tripterygium Wilfordii Hook F*.

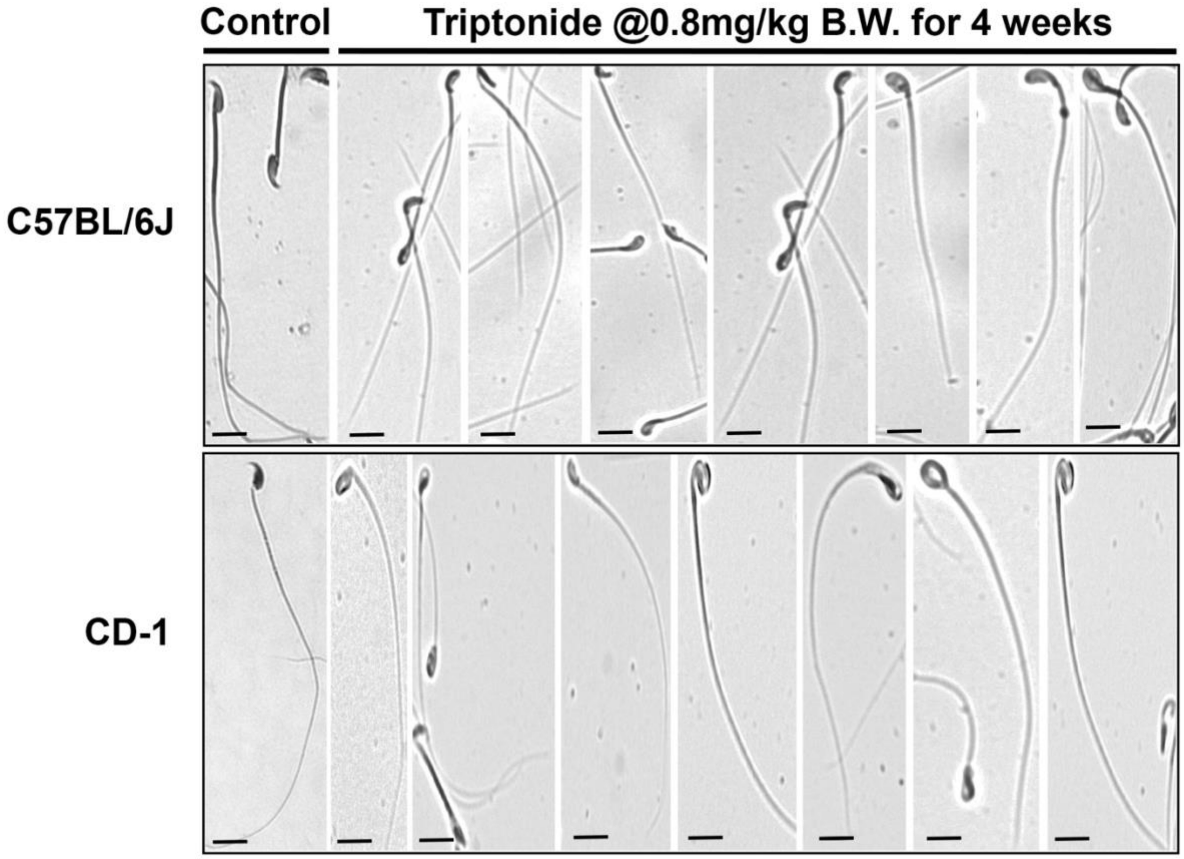

Supplementary Fig. 2 Oral intake of triptonide at single daily doses of 0.8 mg/kg B.W. caused deformed sperm with a close to 100% penetrance after 4 weeks in both adult male C57Bl6/J and CD-1 mice. scale bars = 5 μm.

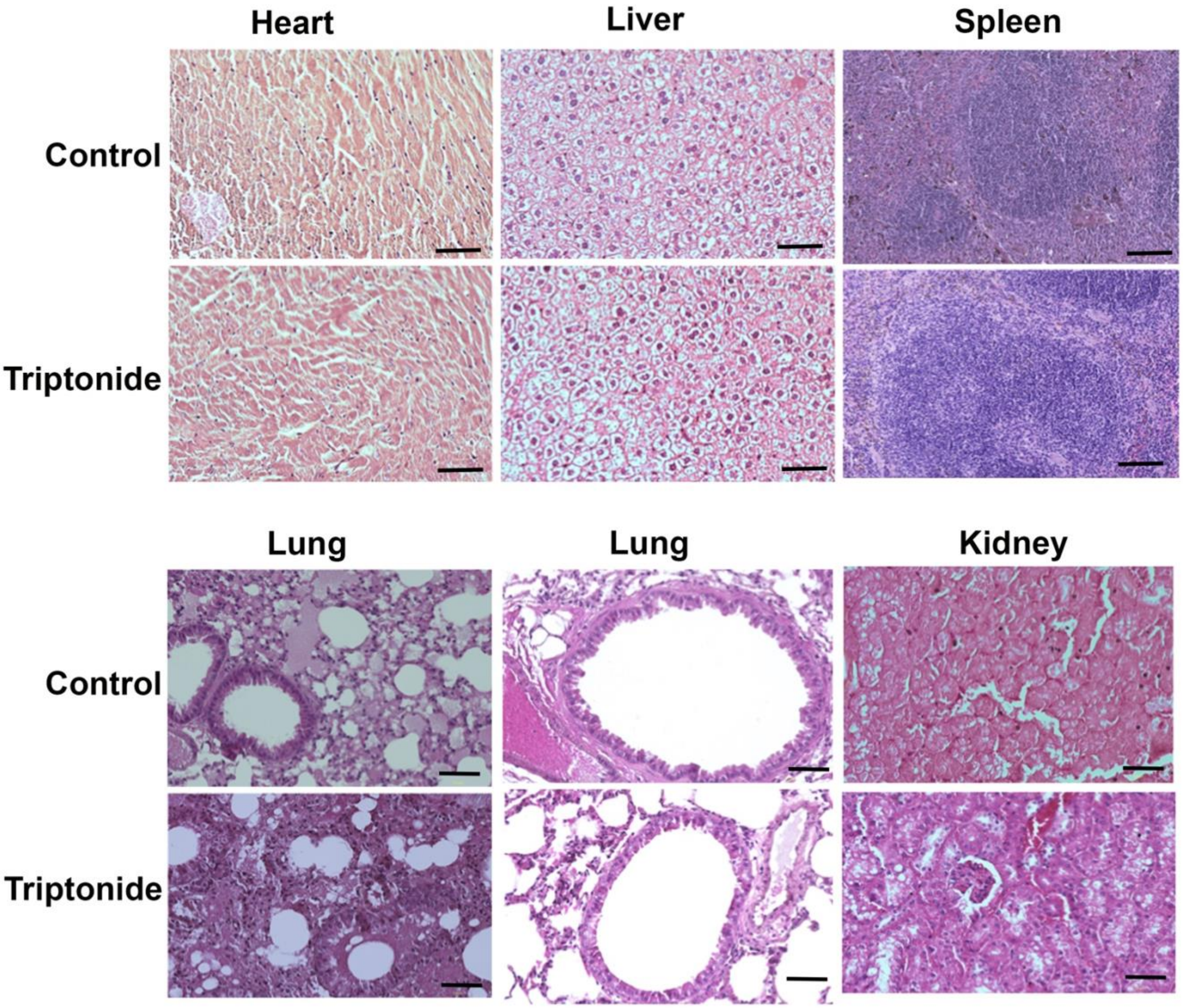

Supplementary Fig. 3 Representative histological images of vital organs including heart, liver, spleen, lung and kidney in adult male C57Bl6/J mice treated with vehicle (control) and triptonide (single daily oral doses at 0.8 mg/kg B.W.) for 4 weeks to induce male infertility. Scale bars = 20 μM.

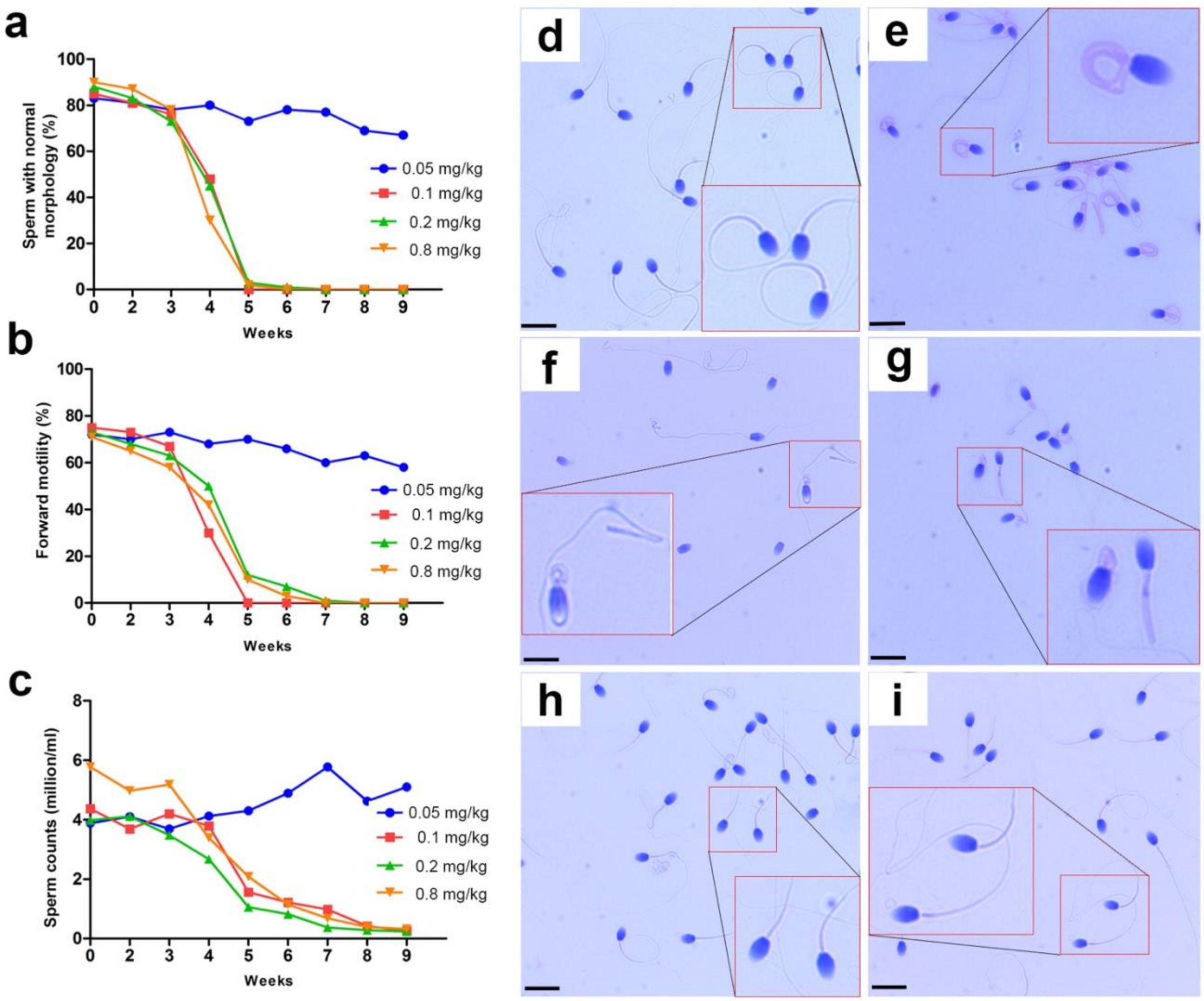

Supplementary Fig. 4 Pilot efficacy testing using four doses of triptonide (0.05, 0.1, 0.2, 0.8 mg/kg B.W.) on four adult male *cynomolgus* monkeys. **a**, Effects of oral intake of triptonide daily on sperm morphology during the 9 week-long treatment. **b,** Effects of oral intake of triptonide daily on sperm forward/progressive motility during the 9 week-long treatment. **c**, Effects of oral intake of triptonide daily on sperm counts during the 9 week-long treatment. **d,** Sperm morphology before treatment using triptonide. **e,** Sperm morphology after 4 weeks of treatment with triptonide (single oral doses at 0.1 mg/kg B.W.). **f,** Sperm morphology after 6 weeks of treatment with triptonide (single oral doses at 0.1 mg/kg B.W.). **g,** Sperm morphology after 8 weeks of treatment with triptonide (single oral doses at 0.1 mg/kg B.W.). **h,** Sperm morphology at week 5 after the cessation of triptonide treatment (single oral doses at 0.1 mg/kg B.W. for 8 weeks). **i,** Sperm morphology at week 8 after the cessation of triptonide treatment (single oral doses at 0.1 mg/kg B.W. for 8 weeks). Scale bars = 20 μM.

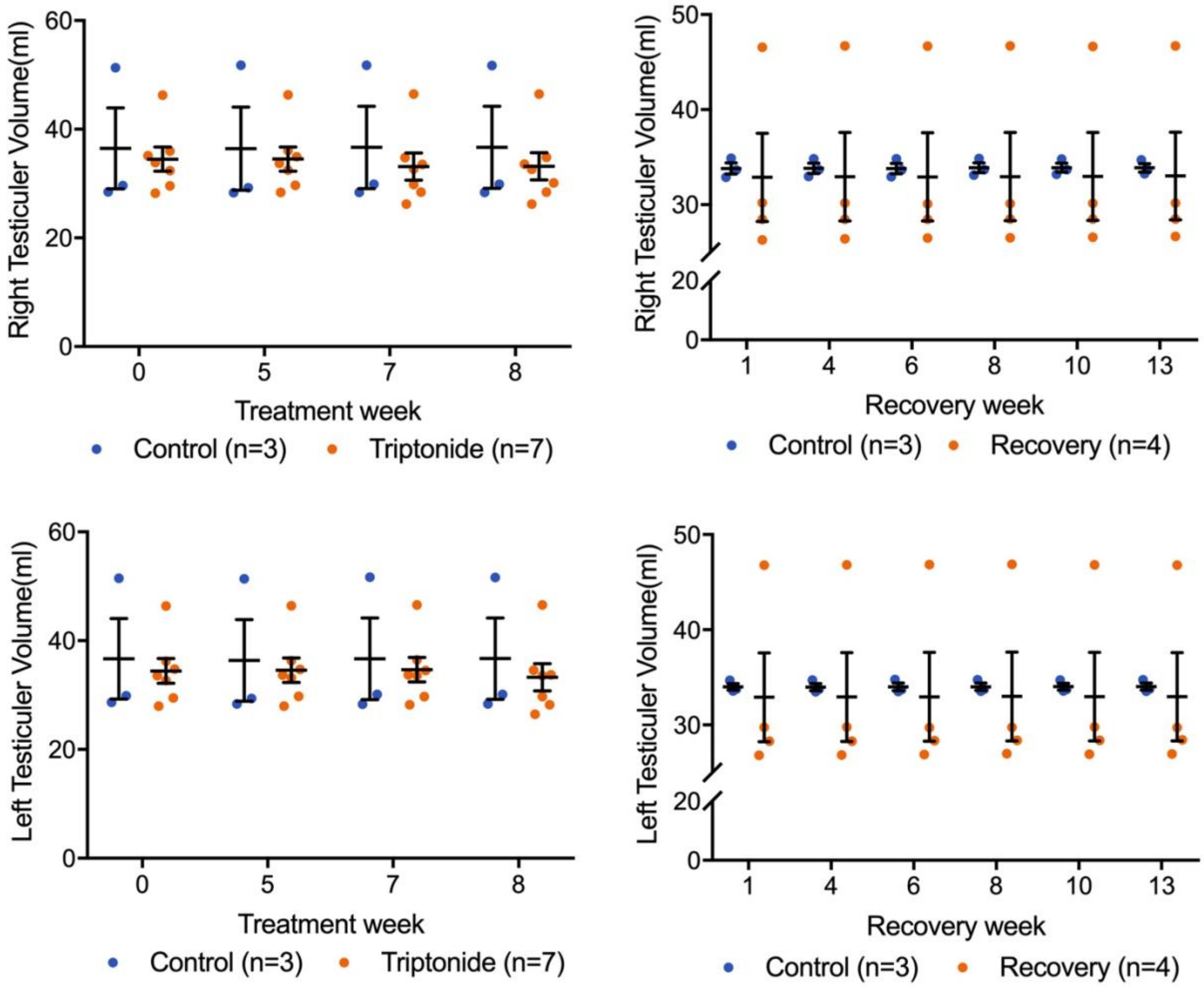

Supplementary Fig. 5 Testis volume in adult male *cynomolgus* monkeys treated with either vehicle (controls, n=3) or triptonide (single oral doses at 0.1 mg/kg B.W. for 8 weeks, n=7) (left two panels), and in adult male *cynomolgus* monkeys recovering from treatment with either vehicle (controls, n=3) or triptonide (single oral doses at 0.1 mg/kg B.W. for 8 weeks, n=4) (right two panels). Individual data points and mean (measure of center) ± SEM (error bars) are shown. Two-way analyses of variance (ANOVA) with Bonferroni multiple comparison test were used to compare differences between control (n=3) and triptonide-treated (n=7) groups at various timepoints, and adjusted $p<0.05$ was considered statistically significant.

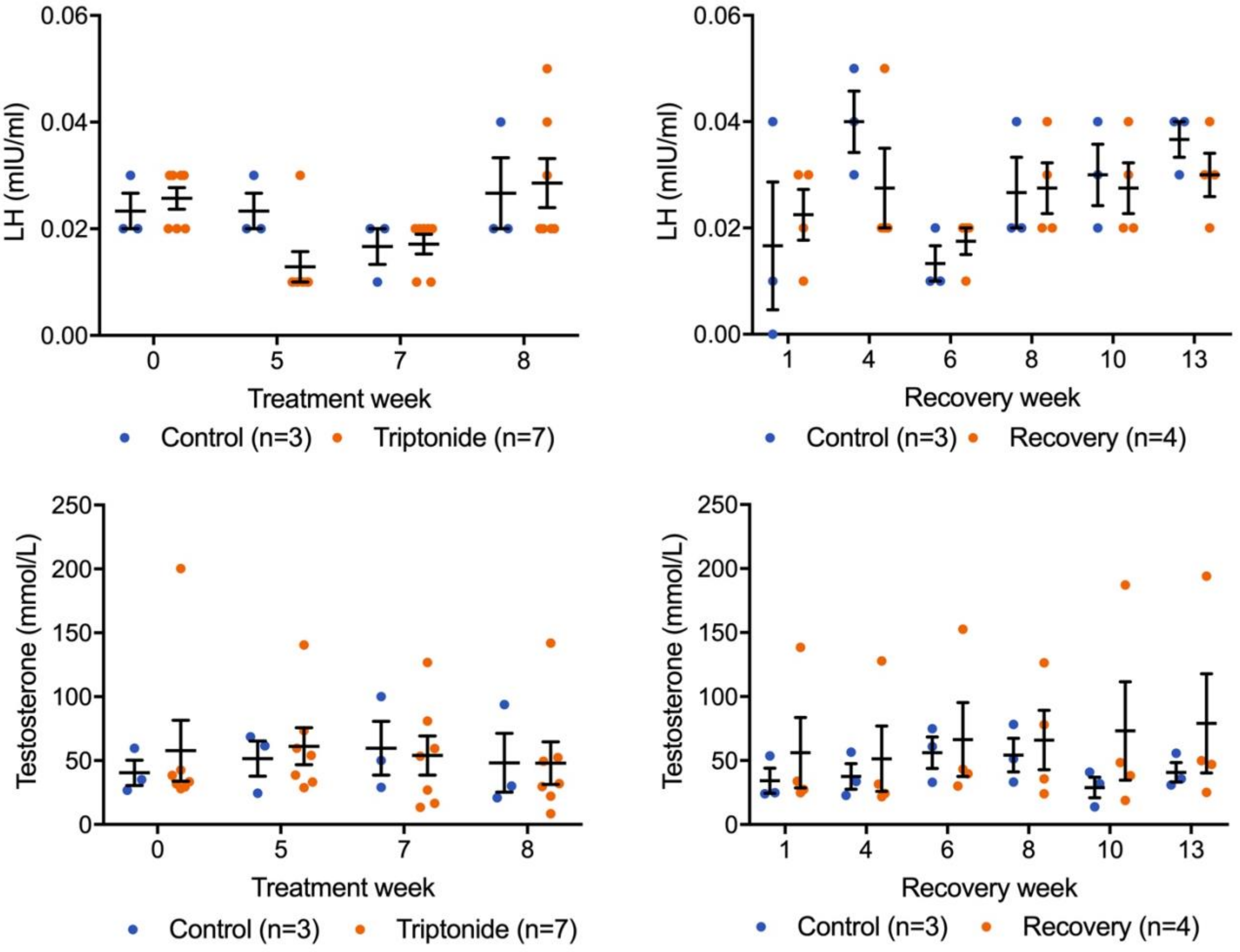

Supplementary Fig. 6 Levels of LH and serum testosterone in adult male *cynomolgus* monkeys treated with either vehicle (controls, n=3) or triptonide (single oral doses at 0.1 mg/kg B.W. for 8 weeks, n=7) (Left two panels), and in adult male *cynomolgus* monkeys recovering from treatment with either vehicle (controls, n=3) or triptonide (single oral doses at 0.1 mg/kg B.W. for 8 weeks, n=4) (Right two panels). Individual data points and mean (measure of center) ± SEM (error bars) are shown. Two-way analyses of variance (ANOVA) with Bonferroni multiple comparison test were used to compare differences between control (n=3) and triptonide-treated (n=7) groups at various timepoints, and adjusted $p<0.05$ was considered statistically significant.

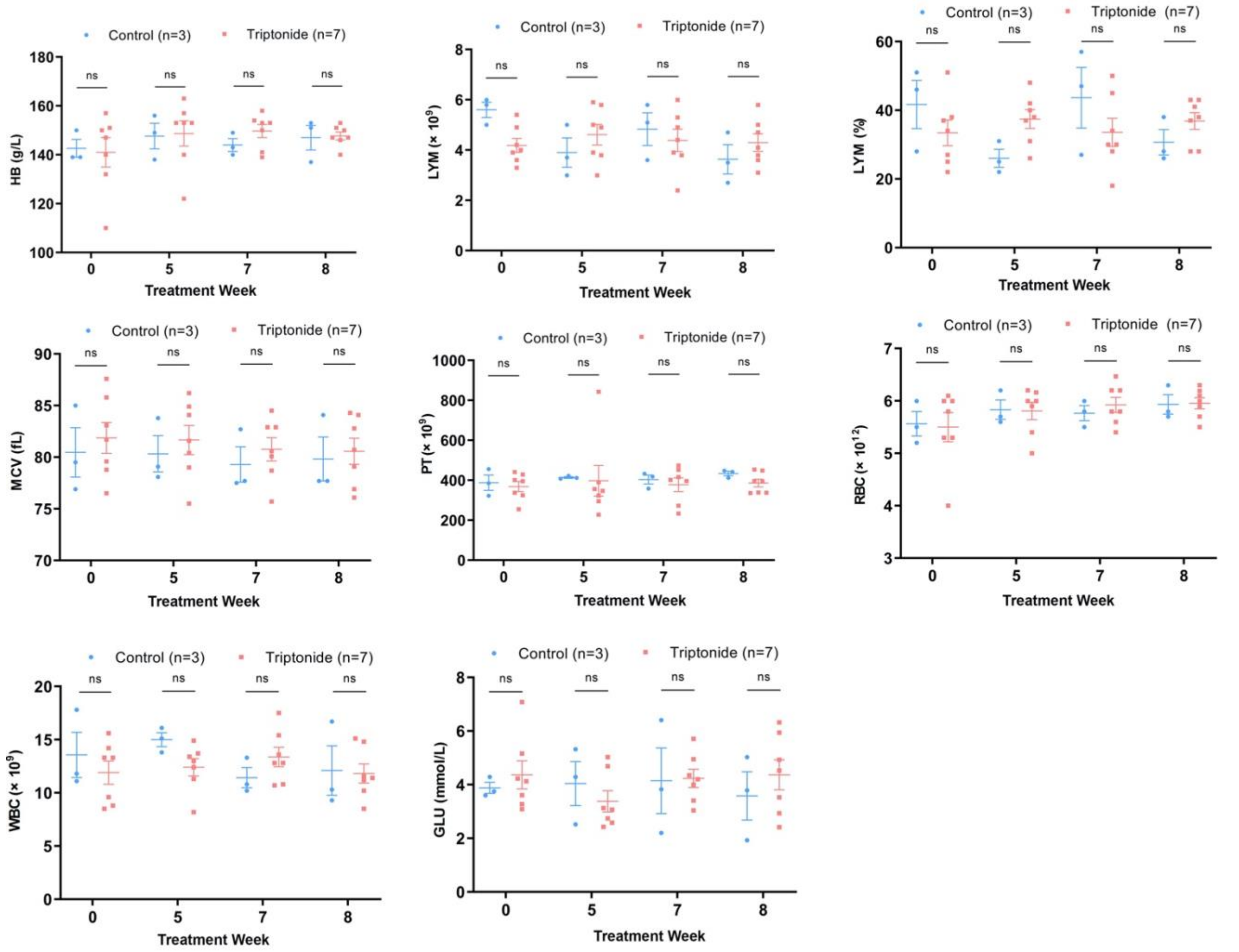

Supplementary Fig. 7 Blood cell counts and chemistry in adult male *cynomolgus* monkeys treated with either vehicle (controls, n=3) or triptonide (single oral doses at 0.1 mg/kg B.W. for 8 weeks, n=7). Parameters measured include counts of the red blood cell (RBC), white blood cell (WBC), and lymphocytes (LYM), percentage of lymphocytes (LYM%), mean corpuscular volume (MCV), and levels of prothrombin (PT), hemoglobin (HB) and glucose (GLU). Individual data points and mean (measure of center) ± SEM (error bars) are shown. Two-way analyses of variance (ANOVA) with Bonferroni multiple comparison test were used to compare differences between control (n=3) and triptonide-treated (n=7) groups at various timepoints, and adjusted $p<0.05$ was considered statistically significant. ns, no statistical significance.

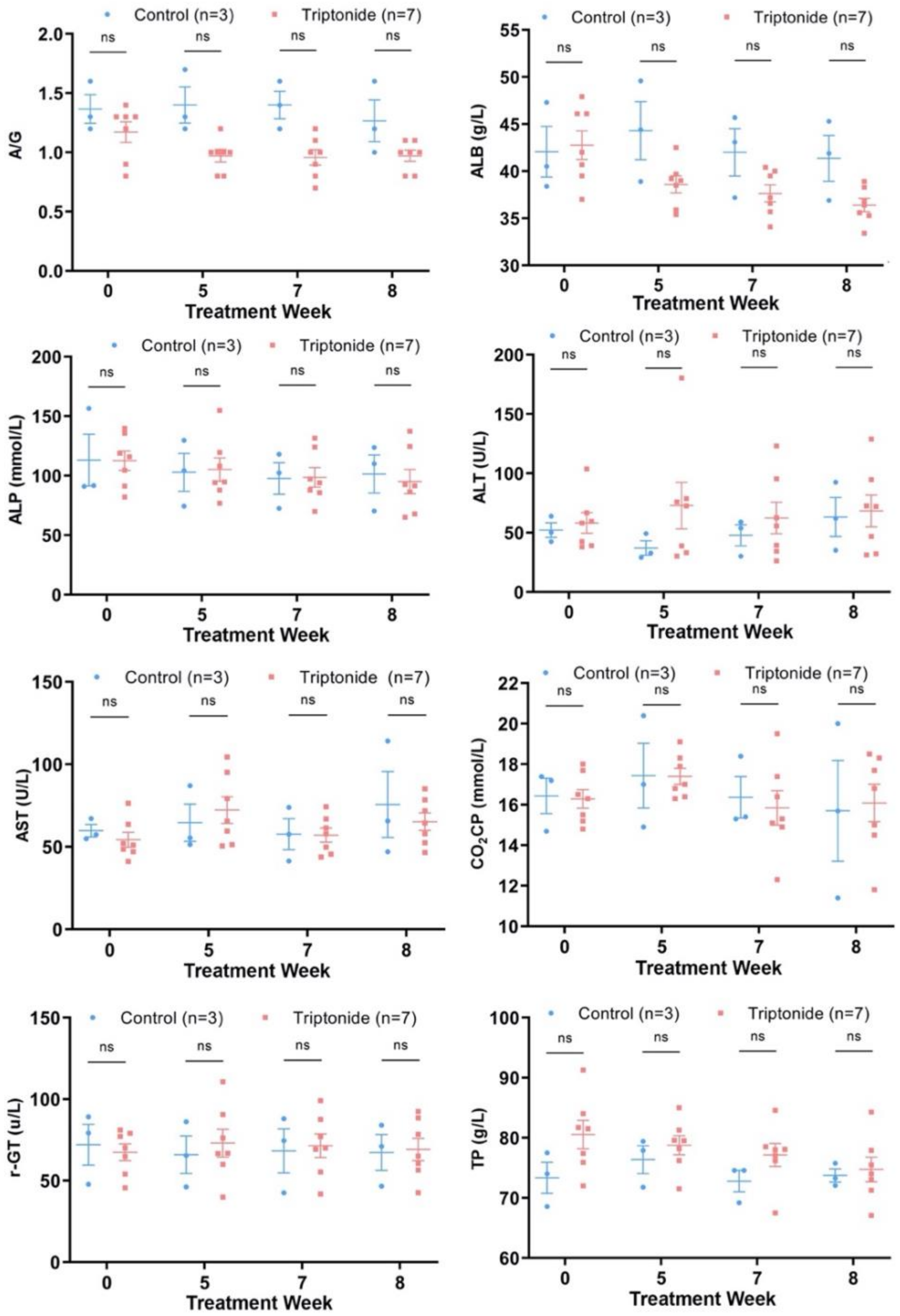

Supplementary Fig. 8 Liver panel in adult male *cynomolgus* monkeys treated with either vehicle (controls, n=3) or triptonide (single oral doses at 0.1 mg/kg B.W. for 8 weeks, n=7). Liver panel contained albumin-globulin (AG), albumin (ALB), alkaline phosphatase (ALP), alanine aminotransferase (ALT), aspartate aminotransferase (AST), carbon dioxide (CO_2), gamma-glutamyl transferase (GT), total protein (TP). Individual data points and mean (measure of center) ± SEM (error bars) are shown. Two-way analyses of variance (ANOVA) with Bonferroni multiple comparison test were used to compare differences between control (n=3) and triptonide-treated (n=7) groups at various timepoints, and adjusted $p<0.05$ was considered statistically significant. ns, no statistical significance.

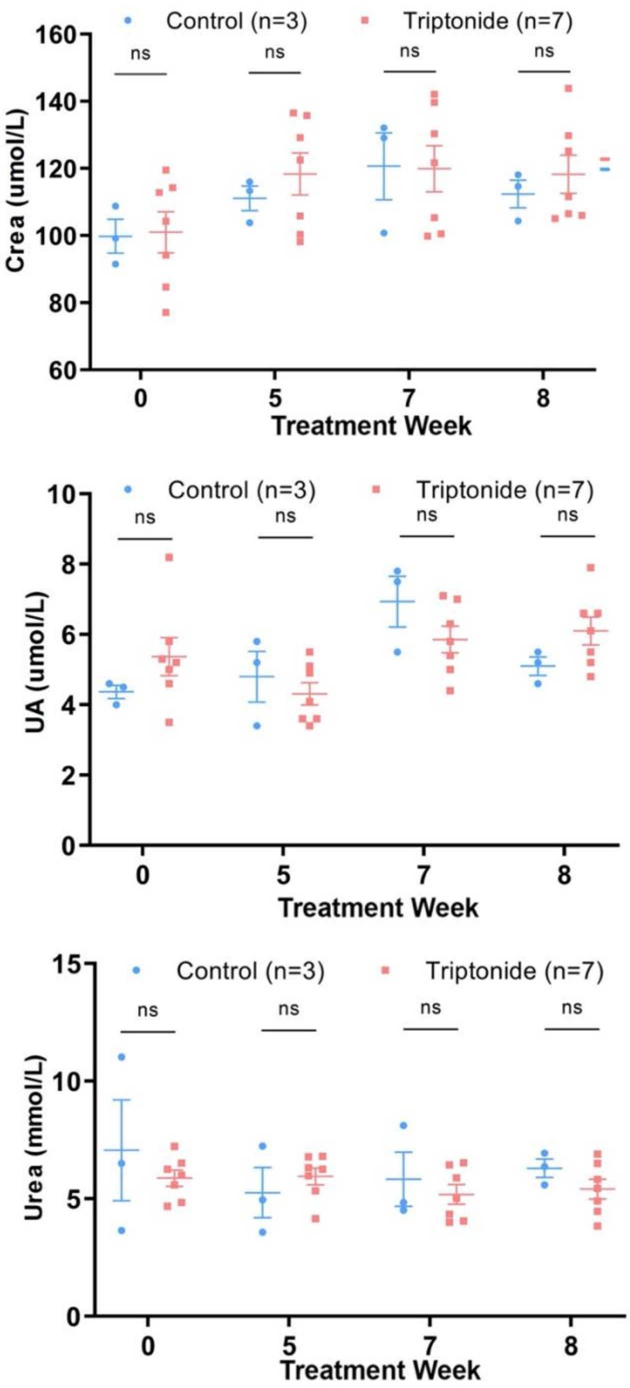

Supplementary Fig. 9 Kidney panel in adult male *cynomolgus* monkeys treated with either vehicle (controls, n=3) or triptonide (single oral doses at 0.1 mg/kg B.W. for 8 weeks, n=7). Kidney panel consisted of creatinine (Crea), urine albumin (UA) and Urea. Individual data points and mean (measure of center) ± SEM (error bars) are shown. Two-way analyses of variance (ANOVA) with Bonferroni multiple comparison test were used to compare differences between control (n=3) and triptonide-treated (n=7) groups at various timepoints, and adjusted $p<0.05$ was considered statistically significant. ns, no statistical significance.

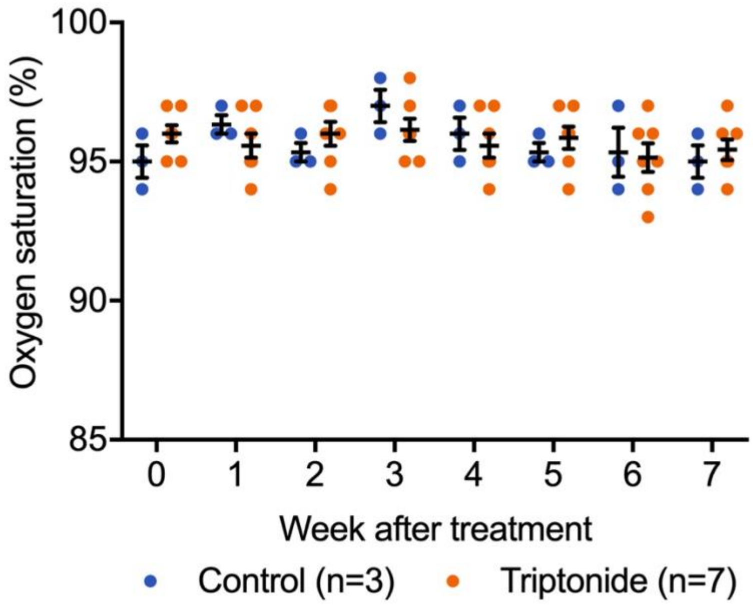

Supplementary Fig. 10 Oxygen saturation (Oxyhemoglobin %) in adult male *cynomolgus* monkeys treated with either vehicle (controls, n=3) or triptonide (single oral doses at 0.1 mg/kg B.W. for 7 weeks, n=7). Individual data points and mean (measure of center) ± SEM (error bars) are shown. Two-way analyses of variance (ANOVA) with Bonferroni multiple comparison test were used to compare differences between control (n=3) and triptonide-treated (n=7) groups at various timepoints, and adjusted $p<0.05$ was considered statistically significant.

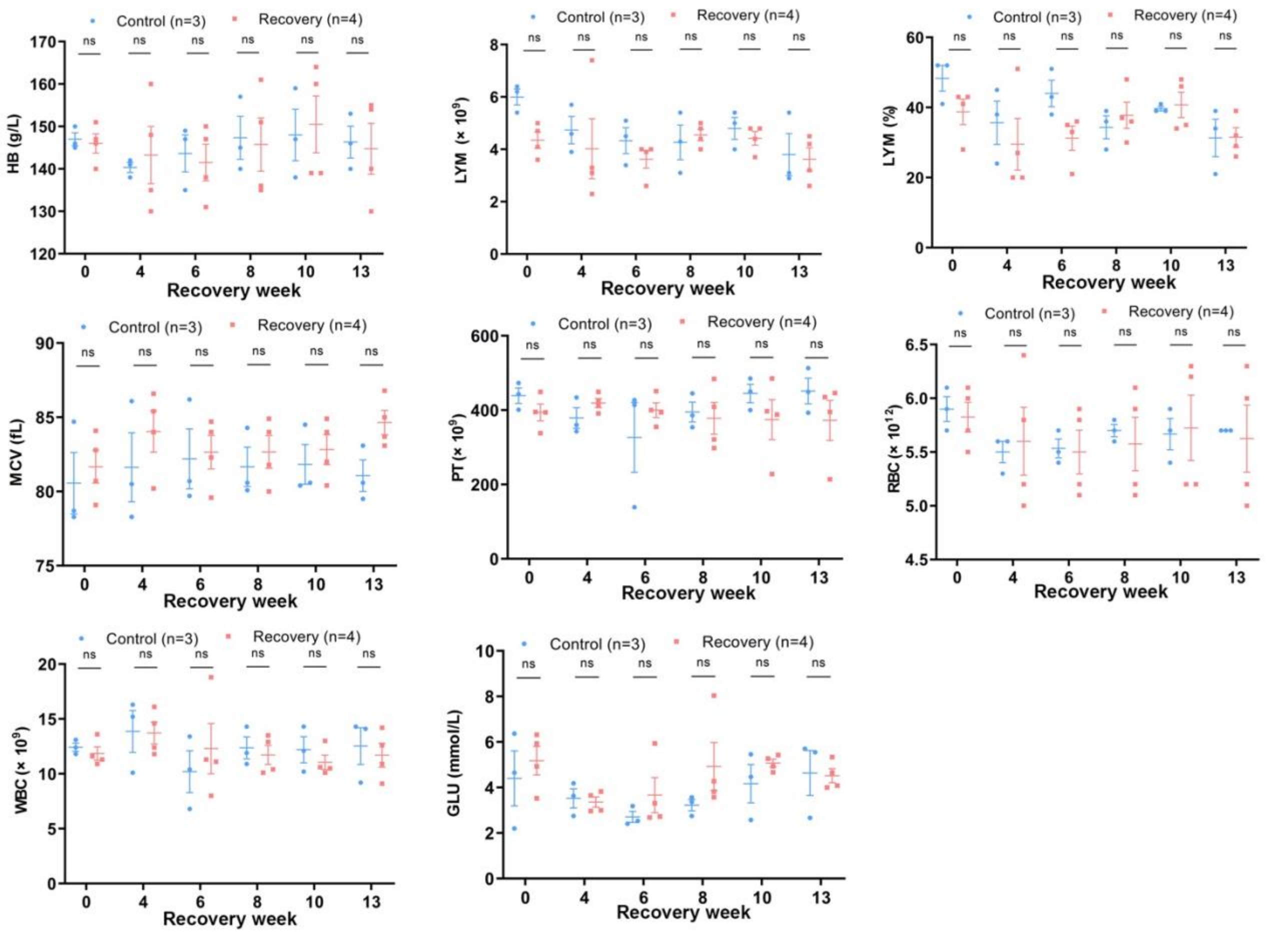

Supplementary Fig. 11 Blood counts and chemistry in adult male *cynomolgus* monkeys recovering from treatment with either vehicle (controls, n=3) or triptonide (single oral doses at 0.1 mg/kg B.W. for 8 weeks, n=4). The x-axis indicates weeks after cessation of triptonide treatment, whereas the y-axis shows parameters measured, including counts of the red blood cell (RBC), white blood cell (WBC), and lymphocytes (LYM), percentage of lymphocytes (LYM%), mean corpuscular volume (MCV), and levels of prothrombin (PT), hemoglobin (HB) and glucose (GLU). Individual data points and mean (measure of center) ± SEM (error bars) are shown. Two-way analyses of variance (ANOVA) with Bonferroni multiple comparison test were used to compare differences between control (n=3) and triptonide-treated (n=7) groups at various timepoints, and adjusted $p<0.05$ was considered statistically significant. ns, no statistical significance.

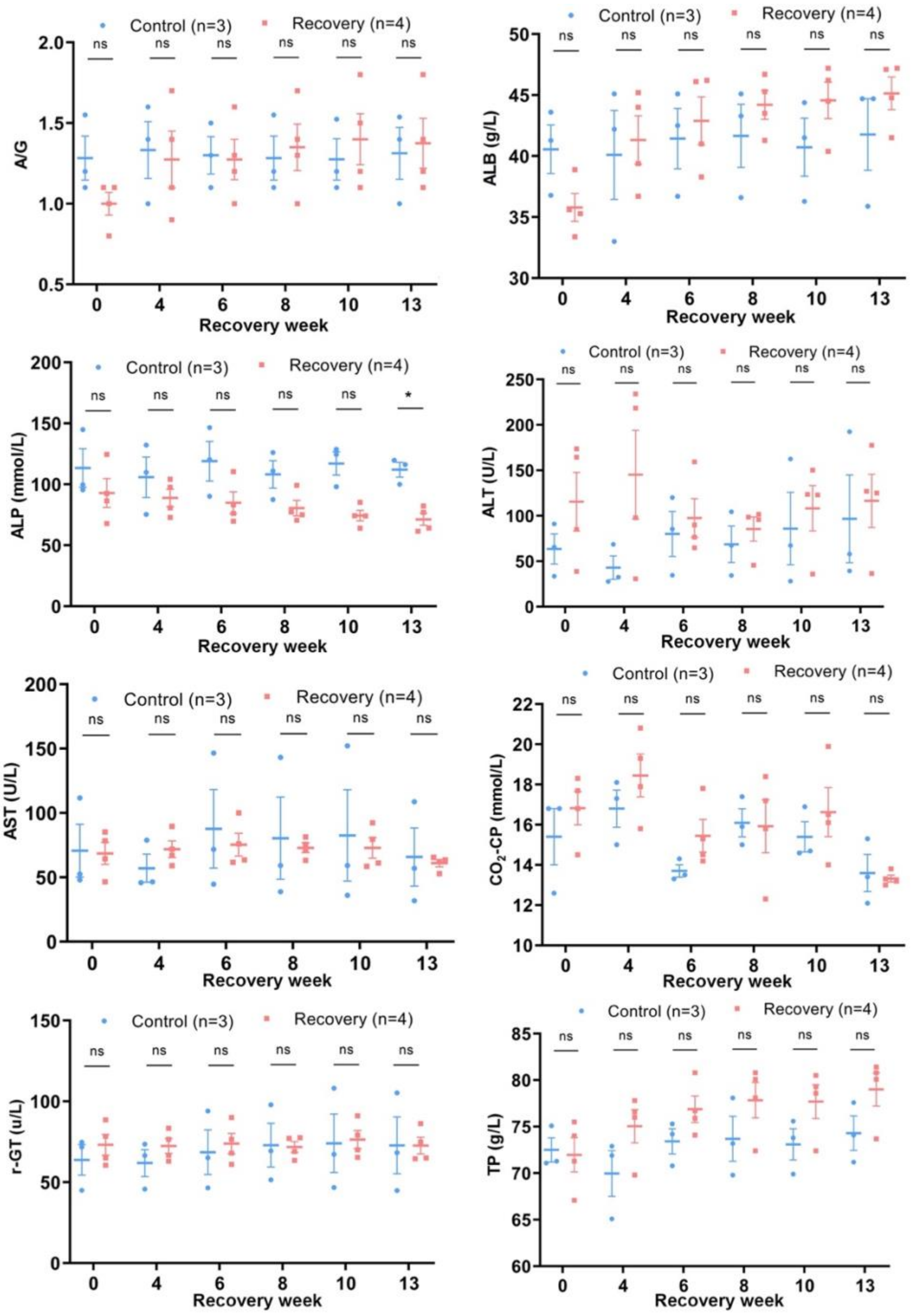

Supplementary Fig. 12 Liver panel in adult male *cynomolgus* monkeys recovering from treatment with either vehicle (controls, n=3) or triptonide (single oral doses at 0.1 mg/kg B.W. for 8 weeks, n=4). The x-axis indicates weeks after cessation of triptonide treatment, whereas the y-axis shows parameters measured, including albumin-globulin (AG), albumin (ALB), alkaline phosphatase (ALP), alanine aminotransferase (ALT), aspartate aminotransferase (AST), carbon dioxide (CO_2), gamma-glutamyl transferase (GT), total protein (TP). Individual data points and mean (measure of center) ± SEM (error bars) are shown. Two-way analyses of variance (ANOVA) with Bonferroni multiple comparison test were used to compare differences between control (n=3) and triptonide-treated (n=7) groups at various timepoints, and adjusted p<0.05 was considered statistically significant. ns, no statistical significance.

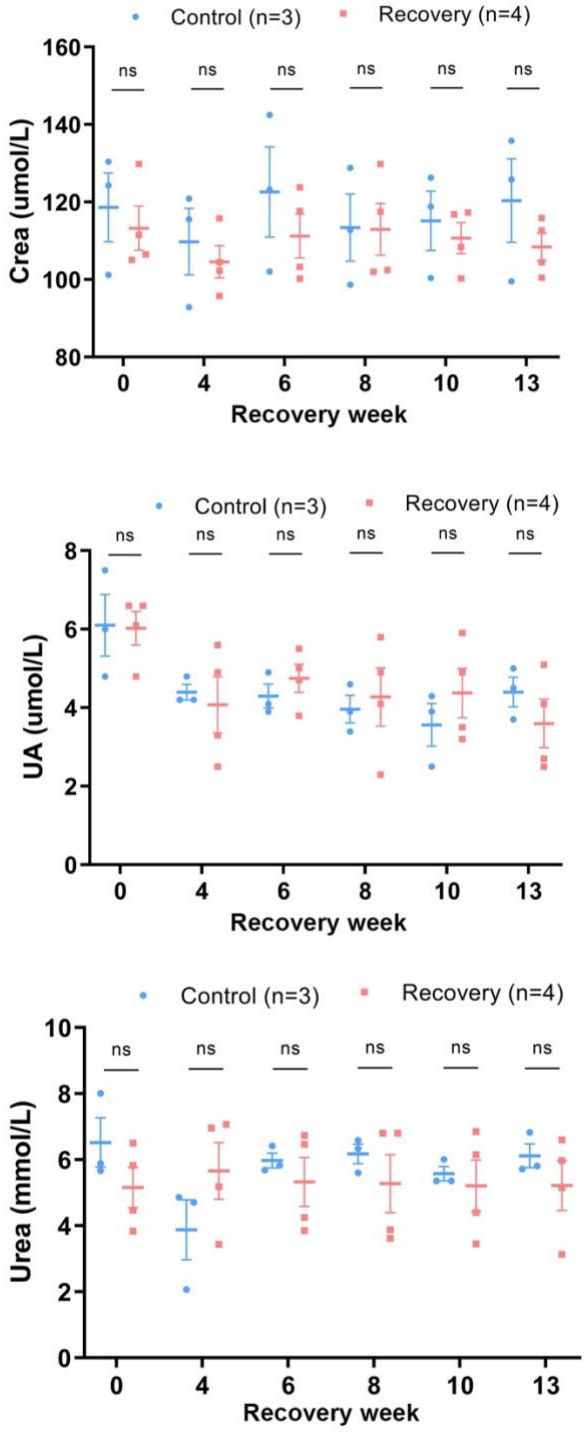

Supplementary Fig. 13 Kidney panel in adult male *cynomolgus* monkeys recovering from treatment with either vehicle (controls, n=3) or triptonide (single oral doses at 0.1 mg/kg B.W. for 8 weeks, n=4). The x-axis indicates weeks after cessation of triptonide treatment, whereas the y-axis shows parameters measured, including creatinine (Crea), urine albumin (UA) and Urea. Individual data points and mean (measure of center) ± SEM (error bars) are shown. Two-way analyses of variance (ANOVA) with Bonferroni multiple comparison test were used to compare differences between control (n=3) and triptonide-treated (n=7) groups at various timepoints, and adjusted $p<0.05$ was considered statistically significant. ns, no statistical significance.

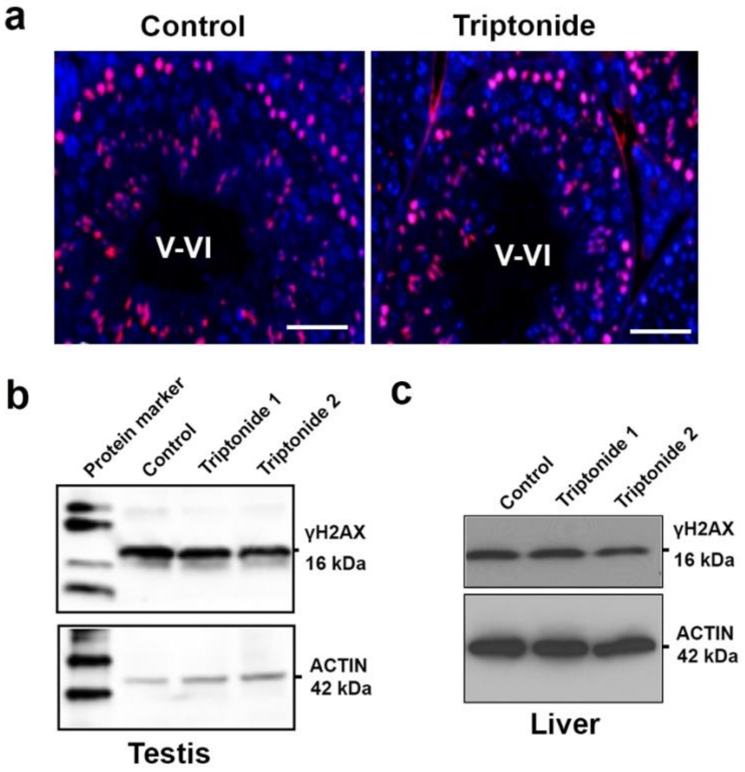

Supplementary Fig. 14 No changes in gamma-H2AX expression in the testes of adult C57BL/6Jmice treated with vehicle (control) and triptonide (single daily oral doses at 0.8 mg/kg B.W.) for 3 months. **a,** Representative immunofluorescent staining of gamma-H2AX in control and triptonide-treated seminiferous tubules at stages V-VI of the epithelial cycle. Scale bars = 20 μM. **b,** A representative Western blot showing levels of gamma-H2AX in the testes of adult C57BL/6Jmice treated with vehicle (control) and triptonide (single daily oral doses at 0.8 mg/kg B.W.) for 4 weeks. Beta-ACTIN was used as loading control. **c,** Representative Western blot results showing levels of gamma-H2AX in the liver of adult C57BL/6J mice treated with vehicle (control) and triptonide (single daily oral doses at 0.8 mg/kg B.W.) for 4 weeks. Beta-ACTIN was used as loading control.

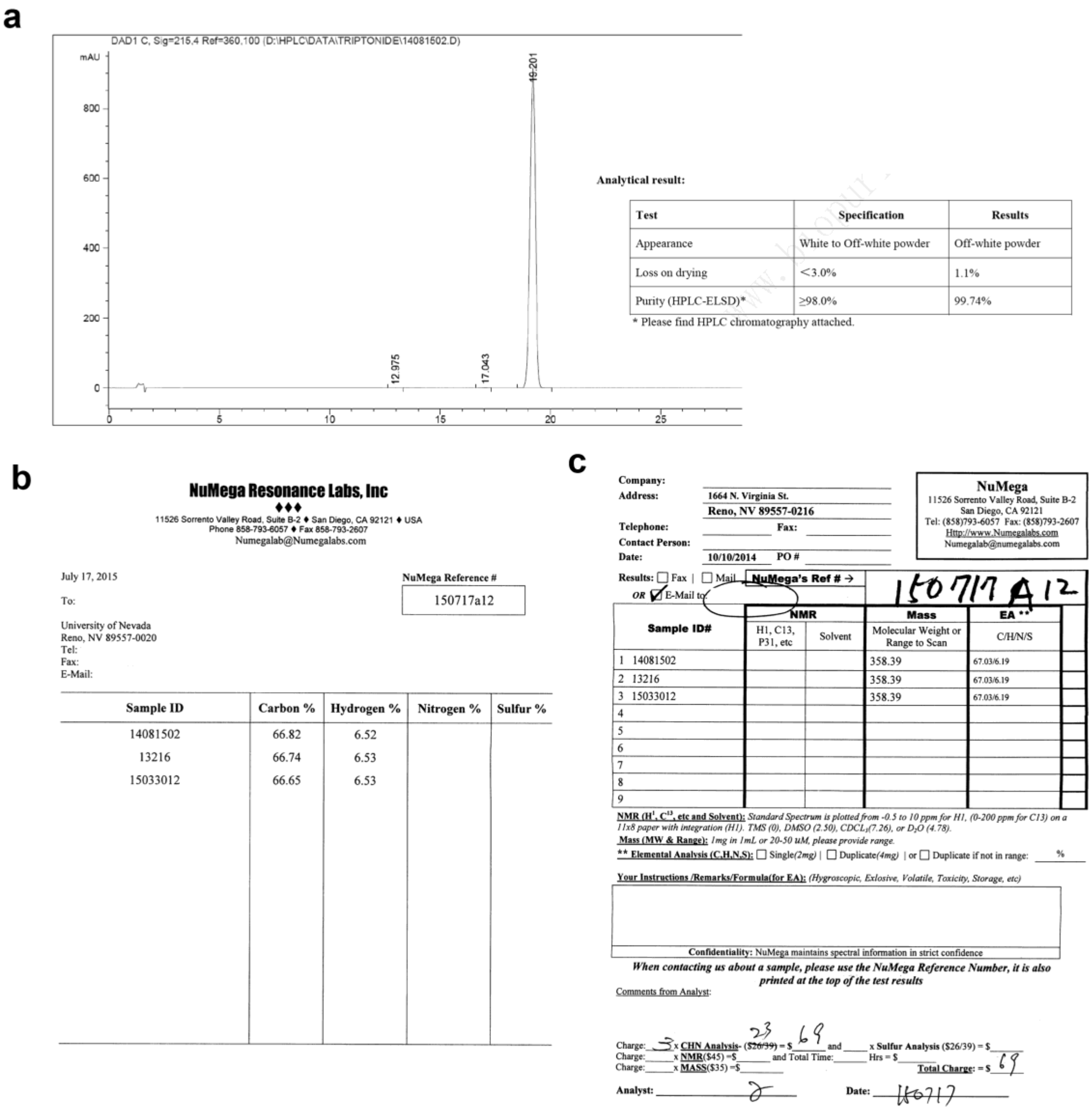

NuMega Resonance Labs, Inc

11526 Sorrento Valley Road, Suite B-2 ♦ San Diego, CA 92121 ♦ USA
Phone 858-793-6057 ♦ Fax 858-793-2607
Numegalab@Numegalabs.com

July 17, 2015

NuMega Reference # 150717a12

To:

University of Nevada
Reno, NV 89557-0020
Tel:
Fax:
E-Mail:

Sample ID	Carbon %	Hydrogen %	Nitrogen %	Sulfur %
14081502	66.82	6.52		
13216	66.74	6.53		
15033012	66.65	6.53		

Company:
Address: 1664 N. Virginia St.
Reno, NV 89557-0216
Telephone: Fax:
Contact Person:
Date: 10/10/2014 PO #

NuMega
11526 Sorrento Valley Road, Suite B-2
San Diego, CA 92121
Tel: (858)793-6057 Fax: (858)793-2607
Http://www.Numegalabs.com
Numegalab@numegalabs.com

Results: ☐ Fax | ☐ Mail
OR ☑ E-Mail to:
NuMega's Ref # → 150717A12

Sample ID#	NMR: H1, C13, P31, etc	NMR: Solvent	Mass: Molecular Weight or Range to Scan	EA **: C/H/N/S	
1 14081502			358.39	67.03/6.19	
2 13216			358.39	67.03/6.19	
3 15033012			358.39	67.03/6.19	
4					
5					
6					
7					
8					
9					

NMR (H^1, C^{13}, etc and Solvent): *Standard Spectrum is plotted from -0.5 to 10 ppm for H1, (0-200 ppm for C13) on a 11x8 paper with integration (H1). TMS (0), DMSO (2.50), $CDCL_3$(7.26), or D_2O (4.78).*
Mass (MW & Range): *1mg in 1mL or 20-50 uM, please provide range.*
**** Elemental Analysis (C,H,N,S):** ☐ Single*(2mg)* | ☐ Duplicate*(4mg)* | or ☐ Duplicate if not in range: ____%

Your Instructions /Remarks/Formula(for EA): *(Hygroscopic, Exlosive, Volatile, Toxicity, Storage, etc)*

Confidentiality: NuMega maintains spectral information in strict confidence

When contacting us about a sample, please use the NuMega Reference Number, it is also printed at the top of the test results

Comments from Analyst:

Charge: 3 x **CHN Analysis**- ($26/39) 23 = $ 69 and ____ x **Sulfur Analysis** ($26/39) = $____
Charge: ____ x **NMR**($45) =$____ and Total Time:____ Hrs = $____
Charge: ____ x **MASS**($35) =$____ **Total Charge:** = $ 69

Analyst: [illegible] **Date:** 150717

Supplementary Fig. 15 Verification of the purity of triptonide purchased from the Chengdu Biopurity Phytochemicals Ltd. with the claimed purity >98% (a) (Lot#: 15033012 and 14081502), and the MedChem Express (Cas#: 38647-11-9. Lot#:13216) using elemental (b) and NMR (c) analyses in NuMega Resonance Labs, Inc. (San Diego, CA)

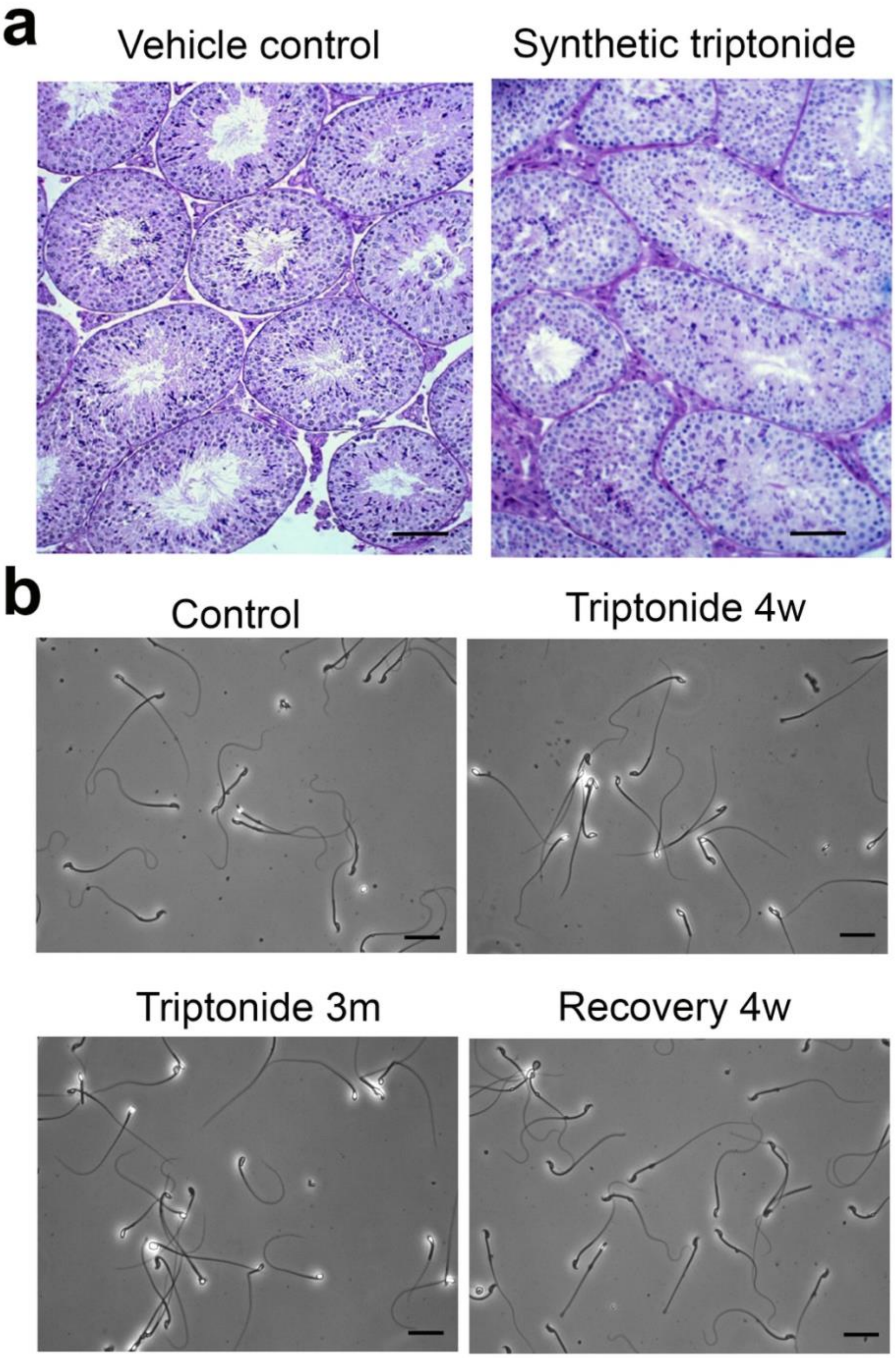

Supplementary Fig. 16 Effects of short- (4 weeks) and long- (3 months) term oral intake of a chemically synthesized triptonide (MedChem Express, Cas#: 38647-11-9, Lot#:13216) at single daily doses of 0.8 mg/kg B.W. on the testes and sperm in adult male C57Bl6/J mice. a, Histology of the testes from male mice treated with vehicle (control) and the synthetic triptonide for 4 weeks. Scale bars = 20 μM. **b,** Morphology of sperm from male mice treated with vehicle (control) or triptonide (0.8 mg/kg B.W. *p.o.* daily) for 4 weeks or 3 months, and those recovered for 4 weeks from 4 weeks of triptonide treatment. Scale bars = 20 μM.

Supplementary Table 1. Preimplantation development of embryos derived from the mouse eggs injected with vehicle- (control) and triptonide-treated sperm (daily oral doses of 0.8 mg/kg B.W. for 4 week).

Sperm of	#Eggs injected with sperm (#Exp.)	#Eggs survived (%)	#Eggs developed *in vitro* into (%):				
			2PN	2-cell	4-cell	Morula	Blastocyst
Control	48 (3)	34 (70.8%)	33 (97.1%)	24 (70.6%)	21 (61.8%)	19 (55.9%)	5 (14.7%)
Triptonide-treated	87 (5)	61 (70.1%)	42 (68.9%)	16 (26.2%)	11 (18%)	7 (11.5%)	3 (4.9%)

Supplementary Table 2. Full-term development of embryos derived from the mouse eggs injected with vehicle- (control) and triptonide-treated sperm (daily oral doses of 0.8 mg/kg B.W. for 4 week).

Sperm of	#Eggs injected with sperm (#Exp.)	#Eggs survived (%)	#2PN (%)	#2PN embryos transferred (#Recipients)	#Live offspring (%)
Control	52 (2)	44 (84.6%)	39 (88.6%)	33 (2)	5 (15%)
Triptonide-treated	53 (2)	41 (77.4%)	29 (70.7%)	29 (2)	0 (0%)

Supplementary Table 3. Summary of data on EC_{50} of triptonide in inducing male infertility in adult C57BL/6J male mice.

Triptonide (mg/kg B.W.) *p.o. daily*	Exp. 1		Exp. 2		Exp. 3		Total number of mice		Pregnant mice	% Pregnancy
	Male	Female	Male	Female	Male	Female	Male	Female		
0	4	12	5	10	5	10	14	32	30	30/32 (94%)
0.1	4	12	5	10	5	10	14	32	13	13/32 (41%)
0.2	4	10	5	10	5	10	14	30	7	7/30 (23%)
0.4	4	12	5	10	5	10	14	32	2	2/32 (6%)
0.8	4	12	5	10	5	10	14	32	0	0/30 (0%)

Supplementary Table 4. General information on the *Cynomolgus* monkeys used in this study.

Number	Age (year)	Weight (kg)	Sex
1	10.9	8.49	male
2	10.6	9.35	male
3	13.0	6.59	male
4	13.0	7.02	male
5	9.0	6.12	male
6	9.5	8.81	male
7	9.3	7.16	male
8	9.1	11.8	male
9	9.5	7.19	male
10	10.2	4.96	male
11	9.8	7.90	male
12	10.4	6.29	male
13	13.2	4.00	female
14	13.2	3.89	female
15	13	3.98	female
16	9.0	4.54	female
17	8.6	4.98	female
18	8.8	5.66	female
