# Supplementary material for: Triptonide is a reversible non-hormonal male contraceptive agent in mice and non-human primates
Source: Nat Commun. 2021 Feb 23;12:1253. doi: 10.1038/s41467-021-21517-5 (PMC7902613; doi:10.1038/s41467-021-21517-5)
Supplement: Supplementary file 15 — Description of Additional Supplementary Files [file 41467_2021_21517_MOESM15_ESM.pdf]

Title: Supplementary Movie 1.
Description: Epididymal sperm collected from control C57BL/6J male mice that received vehicle for 4 weeks.

Title: Supplementary Movie 2.
Description: Epididymal sperm collected from C57BL/6J male mice that received daily single oral doses of triptonide at 0.8 mg/kg B.W. for 4 weeks.

Title: Supplementary Movie 3.
Description: Epididymal sperm collected from C57BL/6J male mice that received daily single oral doses of triptonide at 0.8 mg/kg B.W. for 3 months.

Title: Supplementary Movie 4.
Description: Movie 3. Epididymal sperm collected from C57BL/6J male mice that received daily single oral doses of triptonide at 0.8 mg/kg B.W. for 6 months.

Title: Supplementary Movie 5.
Description: Ejaculated sperm collected from control adult male *cynomolgus* monkeys that received vehicle for 5 weeks.

Title: Supplementary Movie 6.
Description: Ejaculated sperm collected from adult male *cynomolgus* monkeys that received daily single oral doses of triptonide at 0.1 mg/kg B.W. for 5 weeks.

Title: Supplementary Movie 7.
Description: Ejaculated sperm collected from control adult male *cynomolgus* monkeys that received vehicle for 8 weeks.

Title: Supplementary Movie 8.
Description: Ejaculated sperm collected from adult male *cynomolgus* monkeys that received daily single oral doses of triptonide at 0.1 mg/kg B.W. for 8 weeks.

Title: Supplementary Movie 9.
Description: Monkey mating frequency tests.

Title: Supplementary Movie 10.
Description: Epididymal sperm collected from C57BL/6J male mice that recovered for 3 weeks after 4 weeks of triptonide treatment (daily single oral doses of triptonide at 0.8 mg/kg B.W.).

Title: Supplementary Movie 11.
Description: Epididymal sperm collected from C57BL/6J male mice that recovered for 3 weeks after 3 months of triptonide treatment (daily single oral doses of triptonide at 0.8 mg/kg B.W.).

Title: Supplementary Movie 12.
Description: Ejaculated sperm collected from adult male *cynomolgus* monkeys that recovered for 4 weeks after 10 weeks of triptonide treatment (daily single oral doses of triptonide at 0.1 mg/kg B.W.).

Title: Supplementary Movie 13.
Description: Ejaculated sperm collected from adult male *cynomolgus* monkeys that recovered for 6 weeks after 10 weeks of triptonide treatment (daily single oral doses of triptonide at 0.1 mg/kg B.W.).
