# Supplementary material for: Triptonide is a reversible non-hormonal male contraceptive agent in mice and non-human primates
Source: Nat Commun. 2021 Feb 23;12:1253. doi: 10.1038/s41467-021-21517-5 (PMC7902613; doi:10.1038/s41467-021-21517-5)
Supplement: Supplementary file 17 — Reporting Summary [file 41467_2021_21517_MOESM17_ESM.pdf]

nature research

Corresponding author(s): Wei Yan

Last updated by author(s): 01/23/2021

Reporting Summary

Nature Research wishes to improve the reproducibility of the work that we publish. This form provides structure for consistency and transparency in reporting. For further information on Nature Research policies, see our Editorial Policies and the Editorial Policy Checklist.

Statistics

For all statistical analyses, confirm that the following items are present in the figure legend, table legend, main text, or Methods section.

n/a	Confirmed	
☐	☒	The exact sample size (*n*) for each experimental group/condition, given as a discrete number and unit of measurement
☐	☒	A statement on whether measurements were taken from distinct samples or whether the same sample was measured repeatedly
☐	☒	The statistical test(s) used AND whether they are one- or two-sided<br>*Only common tests should be described solely by name; describe more complex techniques in the Methods section.*
☐	☒	A description of all covariates tested
☐	☒	A description of any assumptions or corrections, such as tests of normality and adjustment for multiple comparisons
☒	☐	A full description of the statistical parameters including central tendency (e.g. means) or other basic estimates (e.g. regression coefficient) AND variation (e.g. standard deviation) or associated estimates of uncertainty (e.g. confidence intervals)
☐	☒	For null hypothesis testing, the test statistic (e.g. *F*, *t*, *r*) with confidence intervals, effect sizes, degrees of freedom and *P* value noted<br>*Give P values as exact values whenever suitable.*
☒	☐	For Bayesian analysis, information on the choice of priors and Markov chain Monte Carlo settings
☒	☐	For hierarchical and complex designs, identification of the appropriate level for tests and full reporting of outcomes
☒	☐	Estimates of effect sizes (e.g. Cohen's *d*, Pearson's *r*), indicating how they were calculated

Our web collection on statistics for biologists contains articles on many of the points above.

Software and code

Policy information about availability of computer code

Data collection	No software was used for data collection
Data analysis	Graph Pad Prism 7 (La Jolla, CA, USA) was used for statistical analyses. Proteomics data analyses were conducted using Proteome Discover version 2.1, Sequest (Thermo Fisher Scientific, San Jose, CA, USA; version 2.0.0.802), and Scaffold (version Scaffold 4.8.2, Proteome Software Inc., Portland, OR).

For manuscripts utilizing custom algorithms or software that are central to the research but not yet described in published literature, software must be made available to editors and reviewers. We strongly encourage code deposition in a community repository (e.g. GitHub). See the Nature Research guidelines for submitting code & software for further information.

Data

Policy information about availability of data

All manuscripts must include a data availability statement. This statement should provide the following information, where applicable:

- Accession codes, unique identifiers, or web links for publicly available datasets
- A list of figures that have associated raw data
- A description of any restrictions on data availability

All data, including source data, are available within the Article and Supplementary Files, or available from the corresponding author on reasonable request.

Field-specific reporting

Please select the one below that is the best fit for your research. If you are not sure, read the appropriate sections before making your selection.

- [x] Life sciences
- [ ] Behavioural & social sciences
- [ ] Ecological, evolutionary & environmental sciences

For a reference copy of the document with all sections, see nature.com/documents/nr-reporting-summary-flat.pdf

Life sciences study design

All studies must disclose on these points even when the disclosure is negative.

Sample size	For the pilot mouse efficacy testing, we used 4-6 mice per dosage per time point because we justed wanted to identify effective doses and their corresponding time points. For POC efficacy testing we used >=10 mice per dosage based on Power analyses, which suggested 8. For POC efficacy testing using monkeys, we used 7 adult male monkeys for short-term treatment and reversal experiments and 3 males as vehicle control based on power analyses. The long-term treatment involved 4 instead of 7 adult monkeys due to minimal variation after 8 weeks of treatment. These numbers are close to those suggested by power analyses.
Data exclusions	The mouse POC efficacy testing was performed for a total 5 times with different sample size, ranging from 3-14 mice. Several data points were excluded because of missing doses or other accidents during the treatment, e.g., death due to water line leakage, excessive noise due to constructions near vivarium, etc.
Replication	Both mouse and monkey POC efficacy tests were performed >=3 times with multiple individual animals (n=3-14 for mice, n=3-11 for monkeys). The interval between experiments ranged between 1-6 months. All attempts at replication were successful. We never observed any significant outliers, suggesting high degree of reproducibility.
Randomization	We purchase mice and monkeys in bulk and the animals were randomly selected for different groups receiving various dosages.
Blinding	The mouse POC efficacy testing experiments were conducted multiple times by three different individuals, and we never revealed the name of the chemical to the experimentalists. Since the efficacy testing reported here is proof-of-concept in nature, we did not do blinding, which is only required for determining drug efficacy a clinical trial. However, in our POC efficacy testing on monkeys, the animal care personnel in the primate center administered the chemical, and we only labeled the tubes with S (for SD-1) or V (vehicle) without telling them exactly what were inside the tubes. Therefore, we consider that the monkey data were collected in a somewhat blinding manner.

Reporting for specific materials, systems and methods

We require information from authors about some types of materials, experimental systems and methods used in many studies. Here, indicate whether each material, system or method listed is relevant to your study. If you are not sure if a list item applies to your research, read the appropriate section before selecting a response.

Materials & experimental systems

n/a	Involved in the study	
☐	☒	Antibodies
☒	☐	Eukaryotic cell lines
☒	☐	Palaeontology and archaeology
☐	☒	Animals and other organisms
☒	☐	Human research participants
☒	☐	Clinical data
☒	☐	Dual use research of concern

Methods

n/a	Involved in the study	
☒	☐	ChIP-seq
☒	☐	Flow cytometry
☒	☐	MRI-based neuroimaging

Antibodies

Antibodies used	The primary antibodies used included mouse monoclonal anti-γH2AX (phospS139, Abcam, Cat#: 2635), rabbit polyclonal anti-β-Actin (Abcam, Cat#: 8227), rabbit polyclonal keratin 5 (K5) (BioLegend, Cat#:905501), mouse monoclonal anti-junction plakoglobin/gamma catenine (Life Technologies, Cat#:13-8500). Goat anti-rabbit (SouthernBiotech, Cat#: 4030-05) and goat anti-mouse IgG (H+L) (SouthernBiotech, Cat#: 1036-05) HRP-conjugated secondary antibodies were purchased from SouthernBiotech.
Validation	Mouse monoclonal anti-junction plakoglobin/gamma catenine (Life Technologies, Cat#:13-8500): This ab has been validated by a knockdown experiment in which the ab indeed binds the antigen used for immunization. Moreover, this ab has been used in 17 publications, as shown in the vendor's website (https://www.thermofisher.com/antibody/product/gamma-Catenin-Antibody-clone-PG-11E4-Monoclonal/13-8500).<br>Mouse monoclonal anti-γH2AX (phospS139, Abcam, Cat#: 2635): This ab has been used in 136 publications, as shown in the vendor's website (https://www.abcam.com/gamma-h2ax-phospho-s139-antibody-9f3-ab26350.html?productWallTab=Questions). Our Western and immunofluorescent data, as shown in this study, are consistent with previous data that we published before (PMC5441557, PMC4647214).

Rabbit polyclonal anti-β-Actin (Abcam, Cat#: 8227): We have been using this ab as loading controls for the past 6 years in our lab. The band detected corresponds to the size of beta-ACTIN and this ab has been used in 2227 publications, as documented in the vendor's website (https://www.abcam.com/beta-actin-antibody-ab8227.html).
Rabbit polyclonal keratin 5 (K5) (BioLegend, Cat#:905501): This ab has been used in 98 publications (https://www.biolegend.com/en-us/products/keratin-5-polyclonal-antibody-purified-10956?GroupID=GROUP26). Our Western blot data were consistent with those reported in literature.

Animals and other organisms

Policy information about studies involving animals; ARRIVE guidelines recommended for reporting animal research

Laboratory animals	Male and female adult (2-3 months) mice of C57BL/6J and CD-1 strains were used. Mice were housed in a specific pathogen-free and temperature- and humidity-controlled facility under a light-dark cycle (10h light and 14h dark) with food and water ad libitum at the University of Nevada, Reno. All cynomolgus monkeys used were housed at the Blooming Spring Biological Technology Development Co. LTD, in Guangzhou, China, which is fully accredited by the Association for the Assessment and Accreditations of Laboratory Animal Care International (AAALAC). A total of twelve healthy male adult (9-13 years of age with body weight ranging between 4.96-11.80kg) and six fertility-proven adult females (9-13 years of age with body weight ranging 14 between 3.89-4.00kg) cynomolgus monkeys were used in this study. The general information of the eighteen monkeys used in this study is summarized in Supplemental Table S2.
Wild animals	No wild animals were used in this study.
Field-collected samples	No field-collected samples were used in this study.
Ethics oversight	Animal use protocol was approved by Institutional Animal Care and Use Committee (IACUC) of the University of Nevada, Reno, and is in accordance with the “Guide for the Care and Use of Experimental Animals” established by National Institutes of Health (NIH) (1996, revised 2011). The animal use protocol was approved by the Research Ethics Committee of the Blooming Spring Biological Technology Development Co. LTD.

Note that full information on the approval of the study protocol must also be provided in the manuscript.
